# The Antiobesity Effect and Safety of GLP-1 Receptor Agonist in Overweight/Obese Adolescents Without Diabetes Mellitus: A Systematic Review and Meta-Analysis

**DOI:** 10.7759/cureus.66280

**Published:** 2024-08-06

**Authors:** Nilesh T Katole, Harsh V Salankar, Ajay M Khade, Jyoti S Kale, Nandkishor J Bankar, Punam Gosavi, Bhushan Dudhe, Nishikant Mankar, Obaid Noman

**Affiliations:** 1 Pharmacology, Government Medical College, Nagpur, IND; 2 Pharmacology, NKP Salve Institute of Medical Sciences and Research Centre, Nagpur, IND; 3 Pharmacology, Datta Meghe Medical College, Datta Meghe Institute of Higher Education and Research (Deemed to be University), Nagpur, IND; 4 Physiology, Datta Meghe Medical College, Datta Meghe Institute of Higher Education and Research (Deemed to be University), Nagpur, IND; 5 Microbiology, Jawaharlal Nehru Medical College, Datta Meghe Institute of Higher Education and Research (Deemed to be University), Wardha, IND; 6 Pathology, Datta Meghe Medical College, Datta Meghe Institute of Higher Education and Research (Deemed to be University), Wardha, IND

**Keywords:** adolescent obesity, glp-1ra, glucagon-like peptide-1 receptor agonist, exenatide, semaglutide, childhood obesity, pediatric obesity, meta-analysis, antiobesity effect, overweight/ obese

## Abstract

Background: Glucagon-like peptide-1 receptor agonists (GLP-1 RAs), particularly semaglutide, have become the leading anti-obesity drugs for adults, and a similar trend may follow in adolescents with its recent approval for this age group. However, there is a lack of comparative analysis on the weight loss effects and safety of GLP-1 RAs in obese or overweight pediatric and adolescent populations, especially those who are non-diabetic. This systematic review and meta-analysis aim to provide current evidence on the efficacy and safety of GLP-1 RAs as an anti-obesity treatment in obese or overweight non-diabetic pediatric and adolescent populations.

Method: We searched electronic databases from inception until January 2024 for randomized controlled trials (RCTs) that analyzed the weight loss effect of GLP-1 receptor agonists in adolescents with obesity or overweight without diabetes mellitus. Search results were screened, and eligible studies were included to perform a systematic review and meta-analysis using the Review Manager (RevMan) computer program Version 5.4.1 (The Cochrane Collaboration, 2020) with a random-effects model. The primary efficacy outcomes were changes in body weight, BMI, and BMI Z-score, while the secondary outcomes were the incidence of gastrointestinal adverse events, treatment discontinuation rate due to adverse events, and incidence of serious adverse events. The mean difference, odds ratio, and 95% confidence interval (CI) were used to present the meta-analysis results. Publication bias was visualized using a funnel plot. The quality of the studies was analyzed using Cochrane's Risk of Bias tool (RoB2).

Results: A total of seven RCTs with 576 adolescent participants were included in the analysis. GLP-1 RAs significantly achieved greater weight loss than placebo, with a mean difference of -4.98 kg (-8.49, -1.46), I² = 99%, p = 0.006. Subgroup analysis showed that semaglutide had the most pronounced anti-obesity effect (mean difference of -17.70 kg (-18.89, -16.51), p < 0.00001), compared to liraglutide (mean difference of -2.26 kg (-5.17, 0.65), I² = 99%, p = 0.13) and exenatide (mean difference of -3.17 kg (-4.45, -1.90), I² = 0%, p < 0.0001). Similar results were obtained for other efficacy parameters such as BMI and BMI z-score. However, GLP-1 RA was associated with more gastrointestinal adverse events (such as nausea and vomiting) than placebo (3.06 (2.12, 4.42), I² = 0%, p < 0.00001), with incidence comparable among all GLP-1 RAs in the subgroup analysis. The overall risk of bias among included studies was either of 'some concern' or 'high risk.'

Conclusions: Our meta-analysis demonstrated that GLP-1 RAs had a superior anti-obesity effect compared to placebo or lifestyle modification in obese or overweight non-diabetic adolescents, particularly semaglutide, which had a more pronounced anti-obesity effect than liraglutide and exenatide, with tolerable gastrointestinal adverse effects.

## Introduction and background

The incidence of childhood obesity is rising globally, impacting not only affluent countries but also those with low and middle incomes. It is projected that between 2020 and 2035, the global obesity prevalence will increase for boys from 10% to 20%, and for girls, from 8% to 18% [1]. Obesity in childhood often persists into adulthood, with about two-thirds of children with prepubertal obesity becoming obese adults [2].

In many previous studies, it is established that about half of obese children will remain obese or overweight during adolescence. Similarly, about 80% of obese adolescents will continue to be obese or overweight as adults. And many of them will continue to remain obese until middle age. Therefore, action must be taken to halt and avert childhood obesity. Childhood obesity has emerged as a major public health concern, and it is predicted to add significantly to the chronic disease burden in the coming decades. It is well established in many previous studies that long-term obesity is a risk factor for the development of various chronic illnesses like diabetes mellitus, hyperlipidemia, and essential hypertension in childhood itself and the potential to continue in adult age also [3,4]. Obese or overweight children have to face many psycho-social challenges also children with obesity are more prone to experiencing low self-perception, mental health issues such as depression, academic underperformance, and disordered eating behaviors compared to their peers of healthy weight [5].

Current strategies for treating childhood obesity are multifaceted, focusing mainly on lifestyle changes that involve diet modification and exercise; however, maintaining these changes long-term poses a significant challenge in the pediatric age group. Available pharmacotherapy for childhood obesity is very limited. The approved drugs for childhood obesity included phentermine and liraglutide for those above the 17-year age group. Orlistat is approved for children under 12 years of age [6]. Recently, the FDA approved newer GLP-1 receptor agonists (GLP-1A) semaglutide for adolescent obesity in age groups twelve years and above [7].

GLP-1 receptor analogs have an important function in obesity management. In diabetic patients, they stimulate insulin secretion, lower glucagon secretion, and also promote weight loss. The weight loss effect of GLP-1A is due to reduced calorie intake. This is achieved through decreased gastrointestinal motility and an anorectic effect, which involves activating central GLP-1 receptors in the brain, especially in the arcuate nucleus region [8]. GLP-1 receptors are found in both the pancreas and brain, which contribute to glycaemic control and centrally mediated effects like decreased appetite, increased satiety, and inhibited gastrointestinal motility. While these medications were originally developed for their blood sugar-lowering effects, clinical trials with adults have demonstrated their potential for regulating weight and providing cardiovascular protection [9,10].

While searching for robust evidence of GLP-1 agonists' anti-obesity effects and safety in the pediatric population, we found only a few meta-analyses, which were not up-to-date [11-13]. Consequently, there is a lack of current data on the potential benefits and limitations of GLP-1 agonists in children, especially non-diabetic ones. Thus, a new meta-analysis was planned to evaluate the benefits and limitations of GLP-1 agonists to address obesity in adolescents without diabetes or any other secondary causes of obesity.

## Review

Methods

Criteria for Study Selection

The inclusion criteria of our meta-analysis were as follows: (i) We included only randomized controlled trials (RCTs), (ii) The participants had to be obese (mean age-adapted BMI should be > 30 kg/m^2^) or diagnosed with obesity as defined by the study authors under 18 years old, without diabetes or any other secondary cause of obesity, (iii) The intervention in a study must involve GLP-1 receptor agonists compared to a placebo or standard care (including no treatment or lifestyle modification), (iv) GLP-1 receptor agonists administered through any method (such as oral or injectable) and at any frequency, and (v) Weight-related outcome parameters must be included in studies. The exclusion criteria were if they were duplicates, non-RCTs, reviews, or if involved diabetic (type 1 or type 2) participants, or those with a secondary or syndromic cause of obesity or an age group above 18 years.

Literature Search Strategy

A thorough search in literature databases like PubMed, Scopus, Web of Science, Embase, Science Direct, Wiley Online Library, China National Knowledge Infrastructure (CNKI), and Cochrane Central Register of Controlled Trials (CENTRAL), was carried out for articles published up to January 2024, without any language restrictions.

We also analyzed unpublished research from clinical trial registries (clinicaltrials.gov) and preprint servers (MedRxiv and BioRxiv). The search utilized keywords such as glucagon-like peptide 1, GLP-1, exenatide, liraglutide, semaglutide, pediatric obesity, childhood obesity, and adolescent obesity. We also looked at the list of references of retrieved studies to identify additional relevant publications. search strategy as follows: ("glucagon like peptide 1" OR glp-1 OR GLP-1 OR "Exenatide" OR "Liraglutide"OR "Semaglutide")AND(Pediatrics mostOR child* OR Adolscence OR Adolescents) AND("Obesity"OR"Obese*"OR " overweight")

Measures of Treatment Effects

The following were the outcome parameters of this meta-analysis and systematic review:

Primary Outcomes: Change in body weight, BMI, and BMI z-score for quantitative meta-analysis.

Secondary Outcomes: (i) Incidence of Gastrointestinal adverse events, (ii) Treatment discontinuation rate due to adverse events, and (iii) Incidence of serious adverse events (SAE) or mortality rate.

Data Collection and Analysis

Two authors (JS and PG) independently studied articles for inclusion and exclusion. Any differences of opinion were worked out by discussion or by bringing in a third review author (HS). The research protocol was registered into PROSPERO (International Prospective Register of Systematic Reviews) under the CRD 42020173199 registration number. An adapted Preferred Reporting Items for Systematic Reviews and Meta-Analyses (PRISMA) flow diagram was presented to illustrate trial selection. The main data heads from the included studies were the author's name, publication year, trial identifier, study design, participant details, sample size, interventions, comparators, dosage, and measured outcomes.

Risk of Bias Evaluation

The risk of bias was assessed using the risk of bias tool version 2 (RoB2) developed by Cochrane Collaboration [14]. It was evaluated by two independent authors (AK and NM). If any difference arose, a third reviewer's judgment was used. The bias assessments covered the following heads: (i) generating random sequences, (ii) concealing allocations, (iii) blinding participants, (iv) measurement of blinding outcome, (v) incomplete outcome data if any, (vi) selective outcome reporting, and (vii) other biases. For every judgment, the risk of bias was rated as low, some concern, or high.

Statistical Analysis

We performed a meta-analysis using the Review Manager (RevMan) computer program Version 5.4.1 (The Cochrane Collaboration, 2020) with a random effects model. For the evaluation of the efficacy of GLP-1A on outcome parameters like change in weight or BMI, we used generic inverse variance means difference (MD) with a 95% confidence interval (CI). For the evaluation of safety parameters and dichotomous data analysis, we used odds ratio (OD) with 95% CIs.

Subgroup analyses were performed to explore the effect of specific GLP-1A on change in weight, BMI, BMI z-score reduction, and safety outcome indicators as mentioned above. The Cochrane ‘Q’ statistic was used to estimate statistical heterogeneity, which was quantified with the I² statistic. An I² score of less than 30% was classified as "low," 30-60% as "moderate," 50-90% as "substantial," and 75-100% as significant. P values less than 0.05 were regarded as statistically significant.

Results

Search Results

**Figure 1:**
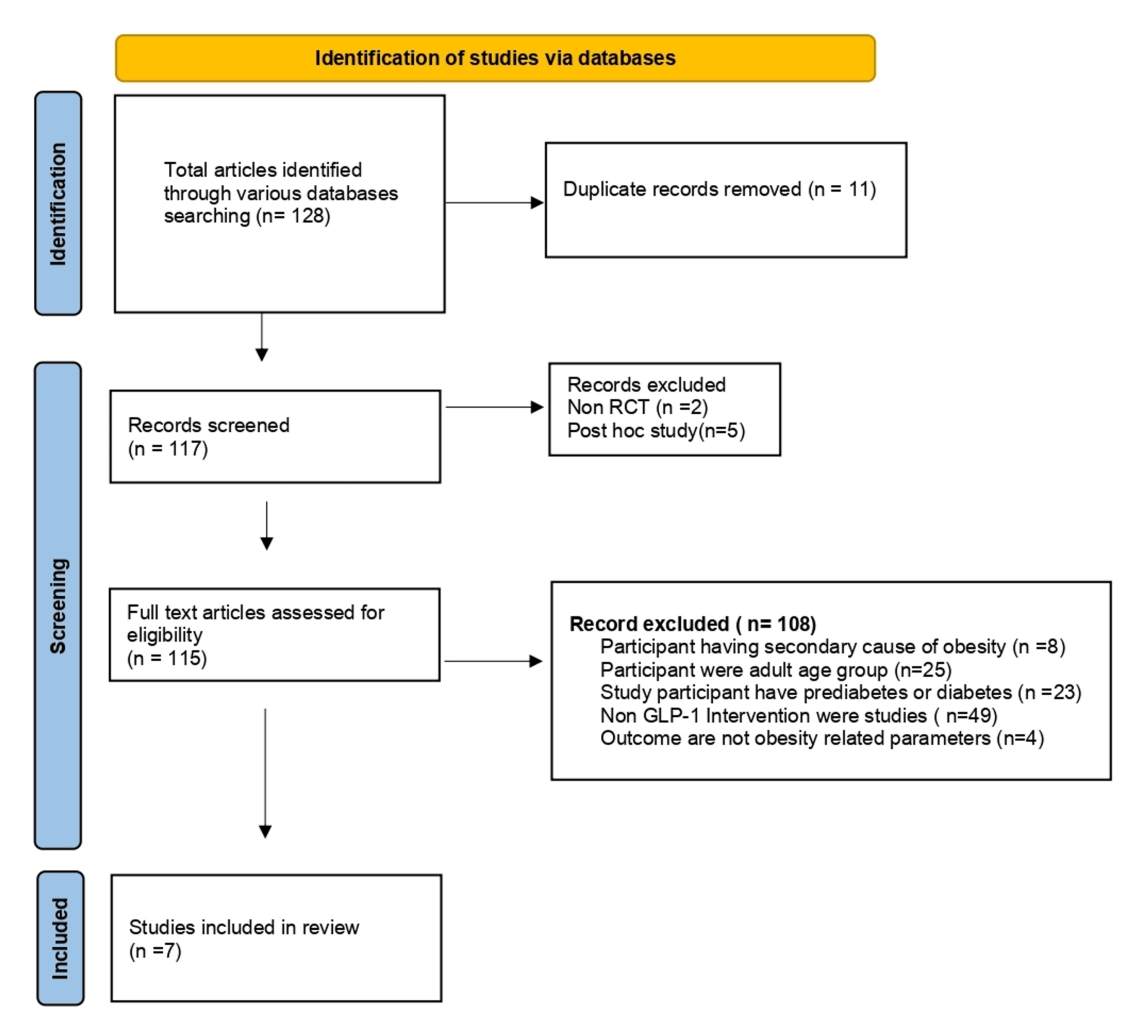
PRISMA flow diagram showing the process of study selection. PRISMA: Preferred Reporting Items for Systematic Reviews and Meta-Analyses

A total of 128 articles were identified through the search strategy as potentially relevant articles, out of which 11 were removed as duplicates. After screening the abstracts of the remaining 117 references, only seven studies were eligible for more detailed evaluation based on predefined inclusion and exclusion criteria. The primary grounds for exclusion were interventions other than GLP-1 agonists, the inclusion of adult participants, participants with secondary causes of obesity, and participants with diabetes, unrelated to obesity. Figure 1 provides a visual representation of the study selection process.

Study Characteristics

The main characteristics of the eligible studies are shown in Table 1. Among the seven studies included, three used exenatide [15-17], three used liraglutide [18-20], and the remaining one used semaglutide [21] as intervention. One study used lifestyle modification as a control [15], and another used a placebo with lifestyle modification as a comparator [18], while the remaining studies used a placebo as a comparator.

**Table 1:** Characteristics of the included studies LM: lifestyle modification; NR: not reported

Sr no.	Study ID	Author and year	Place of study	Intervention	Control	Study design	Sample size, n	Age (years), mean	Target Dose	Duration (weeks)	Weight (Kg), mean±SD	BMI (kg/m2), mean±SD	BMI z-score, mean±SD
1	EXENA2012	Kelly et al. [15] (2012)	United States	Exenatide	LM	Randomized, open-label, crossover, clinical trial	12	12.7	5-10 µg twice daily	12 weeks	93.8±20.6	36.7±4.8	NR
2	EXENA2013	Kelly et al. [16] (2013)	United States	Exenatide	Placebo	Randomized control trial	26	15.2	5-10 µg twice daily	12 weeks	124±19.3	42.5±6.81	NR
3	EXENA2020	Weghuber et al. [17] (2020)	Sweden, Austria	Exenatide	Placebo	Randomized control trial	44	14.5	2 mg/week	24 weeks	104.1±20.5	36.1±4.9	3.2±0.6
4	LIRA2017	Danne et al. [18] (2017)	Germany	Liraglutide + LM	Placebo + LM	Randomized control trial	21	15.1	3 mg/ day	5 weeks	103.5±12.8	36.5±3.7	3.17±0.49
5	LIRA2018	Mastrandrea et al. [19] (2018)	United States	Liraglutide	Placebo	Randomized control trial	24	9.9	3 mg/ day	8 weeks	71.5 ±15.4	44.3±4.1	3.9±0.9
6	LIRA2020	Kelly et al. [20] (2020)	United States, Belgium, Mexico, Russia, Sweden	Liraglutide	Placebo	Randomized control trial	251	14.6	3 mg/ day	56 weeks	102.2±21.6	35.3±5.1	3.14±0.65
7	SEMA2022	Weghuber et al. [21] (2022)	Austria, United Kingdom, United States, Belgium	Semaglutide	Placebo	Randomized control trial	201	15.4	2.4 mg/week	75 weeks	107.5±24.5	37.0±6.4	3.31±0.86

Out of these seven studies, three were carried out in the United States, two in Germany, and the other two were international consortia involving sites in countries like Russia, Sweden, Mexico, and the United Kingdom.

Six studies were parallel RCTs, and one used a crossover RCT design. A total of 576 children and adolescent participants have been included in this meta-analysis and among these 333 participants received GLP-1 analogs. Most of the studies were of a short duration ranging from five to 24 weeks and had small sample sizes ranging from 11 to 44 participants, except for two studies: one that evaluated liraglutide over 56 weeks with 251 participants [20] and another that evaluated semaglutide over 75 weeks with 201 participants [21]. Participants' average age was 13.7±2.16 years and there was a slight female predominance (53.3%).

The baseline mean weights of participants ranged from 71.5 kg to 124 kg, with BMIs of 35.3-44 kg/m² and BMI z-scores of 3.14-3.9. In exenatide studies, a dose of exenatide was 5-10 mcg per day, and one exenatide study by Weghuber et al. used a 2 mg per week dose [17]. Liraglutide started with 0.3 mg and gradually raised to 3 mg per day maximum based on participant tolerability in all three liraglutide studies. Semaglutide was administered at a dose of 2.4 mg once a week in a semaglutide trial.

Efficacy Parameters

**Figure 2:**
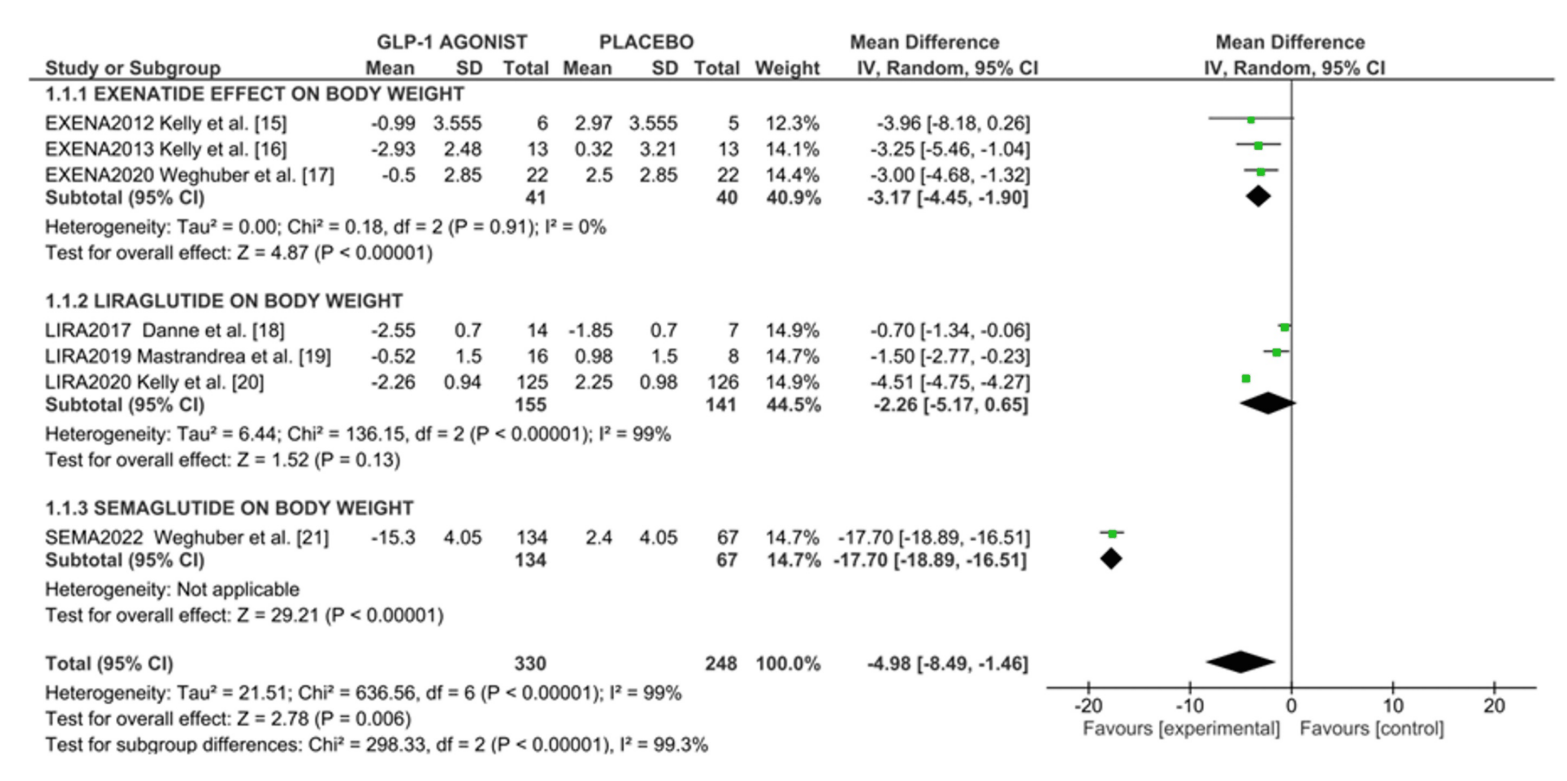
Forest plot of weight loss effect of GLP-1 agonist in overweight/obese adolescents. References: [15-21]

Change in body weight: All seven RCTs reported the effect of GLP-1 analogs against weight change. A random effects model of meta-analysis was conducted, involving 333 adolescents assigned to GLP-1R agonists and 248 assigned to control groups. The mean reduction in body weight with the highest dose of GLP-A ranged from -15.3 kg to -0.5 kg. The pooled data analysis showed that GLP-1R agonists moderately decreased body weight compared to a control group, as indicated in the forest plot (Figure 2) with a mean difference of -4.98 kg (95%CI -8.49, -1.46), I² = 99%, p = 0.006. The overall heterogeneity was at 99%.

As shown in Figure 2, we conducted a subgroup analysis as per intervention regimes. Pooled data analysis showed exenatide reduced body weight loss of -3.17 kg (95%CI -4.45, -1.90), I^2^ = 0%, p=0.0001 and liraglutide showed little or no change in body weight -2.26 (95%CI -5.17, 0.65), I^2^ = 99% p=0.13; however, semaglutide shows highest reduction in body weight (-15.3 kg) as compared to the control group (2.4 kg) with mean difference of -17.70 (95%CI -18.89, -16.51) p<0.00001.

**Figure 3:**
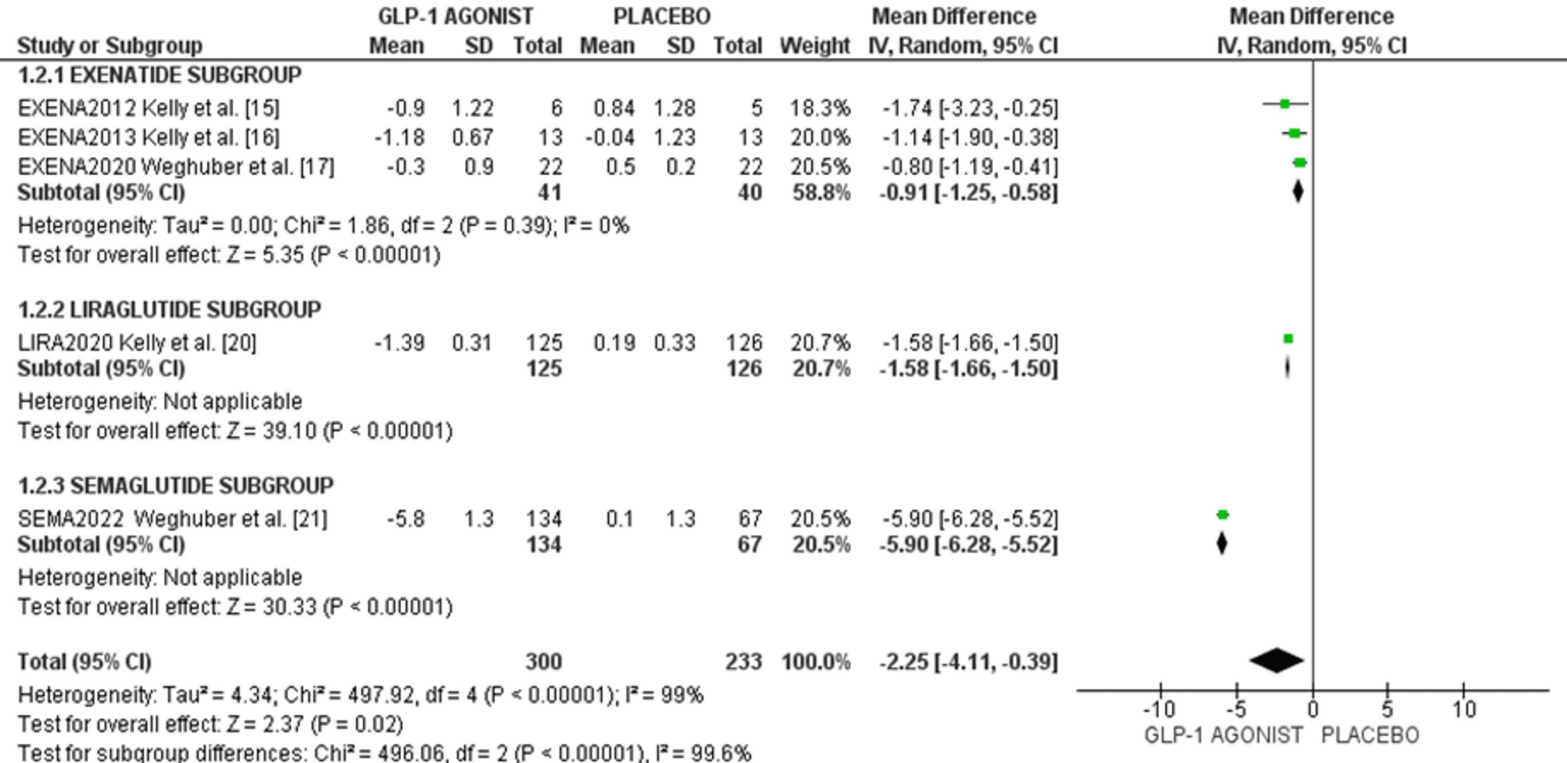
Forest plot of effects of GLP-1 agonist on BMI outcome in overweight/obese adolescents. References: [15-17,20,21]

Effect on change in BMI: Five RCTs involving a total of 533 participants reported efficacy parameters in terms of BMI. Pooled data analysis as displayed in the forest plot (Figure 2b) showed GLP-1RA recipients showed an overall BMI reduction of -2.25 kg/m² (95%CI -4.11 to -0.39), I² = 0%, compared to placebo, which is statistically significant. The highest BMI reduction was observed with semaglutide (-5.90 kg/m² (95%CI -6.28, -5.52)), followed by liraglutide (-1.58 kg/m² (95%CI -1.66, -1.50)), and lastly exenatide (-0.91 kg/m² (95%CI -1.25, -0.58)) as shown in the forest plot in Figure 3.

**Figure 4:**
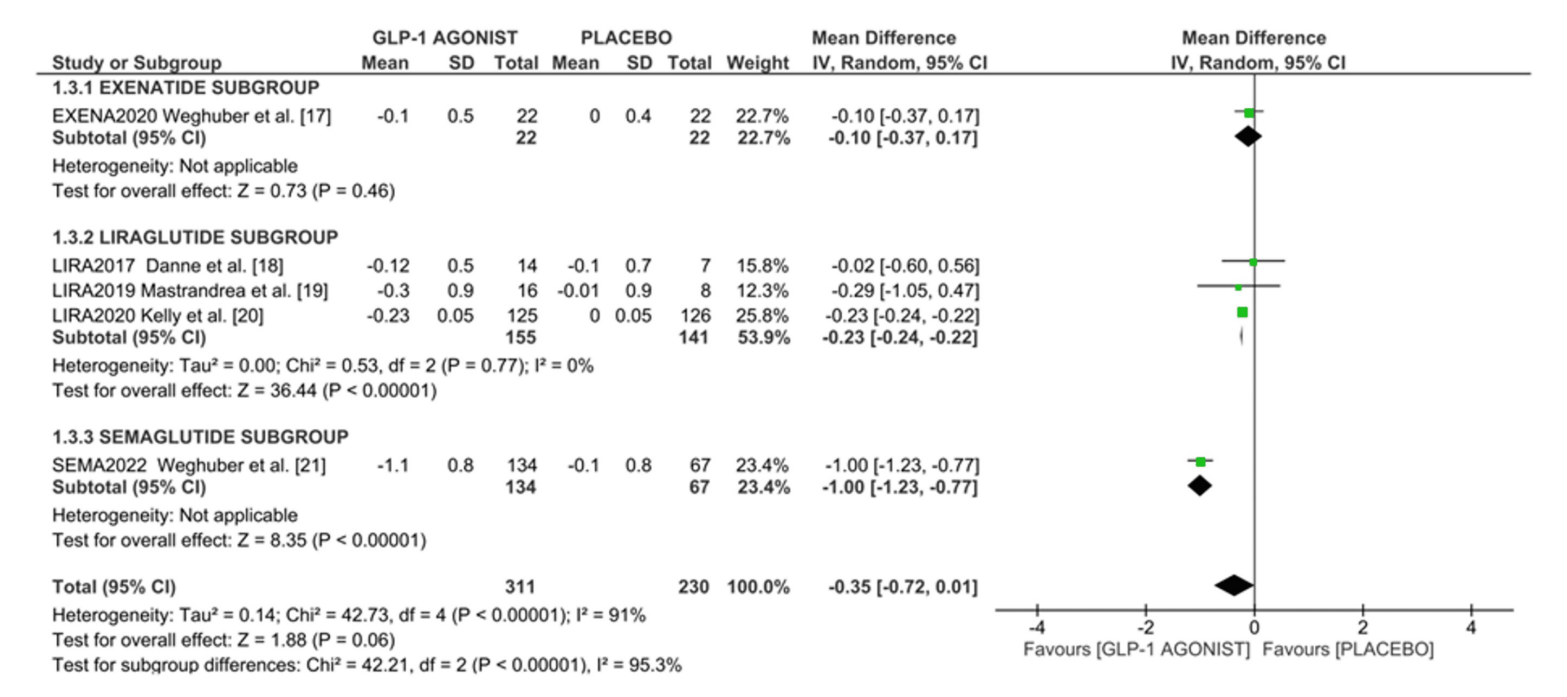
Forest plot of the effect of GLP-1 agonist on BMI z-score in overweight/obese adolescents. References: [17-21]

Effect on BMI z-score: Five studies, totaling 540 participants reported the efficacy of GLP-1A in terms of BMI z-score. Pooled data analysis showed that GLP-1 agonists reduced BMI z-scores by -0.35 (95%CI -0.72, -0.01), p < 0.00001. The highest reduction in BMI z-score was observed with semaglutide (-1.00 (95%CI -1.23, -0.77)), followed by liraglutide (-0.23 (95%CI -0.24, -0.22)), and lastly, exenatide (-0.10 (95%CI -0.37, 0.17)) as shown in the forest plot in Figure 4.

Safety Parameters

We analyzed safety outcome measures with OR with random effect model.

**Figure 5:**
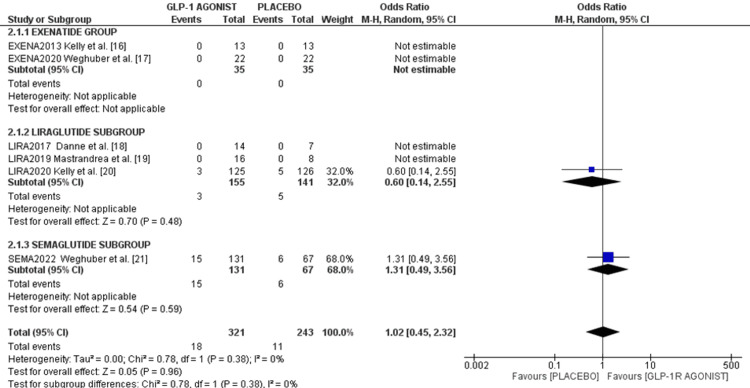
Forest plot of incidence of gastrointestinal adverse events due to GLP-1 agonist in obese adolescents. References: [16-21]

Incidence of gastrointestinal adverse events: GLP-1R agonists were associated with an enhanced risk of gastrointestinal adverse events when compared with the control group with OR of 3.06 (95%CI 2.12, 4.42), I² = 0%, p < 0.00001. The incidence was comparable among all GLP-1R agonists in the subgroup analysis as shown in the forest plot in Figure 5.

**Figure 6:**
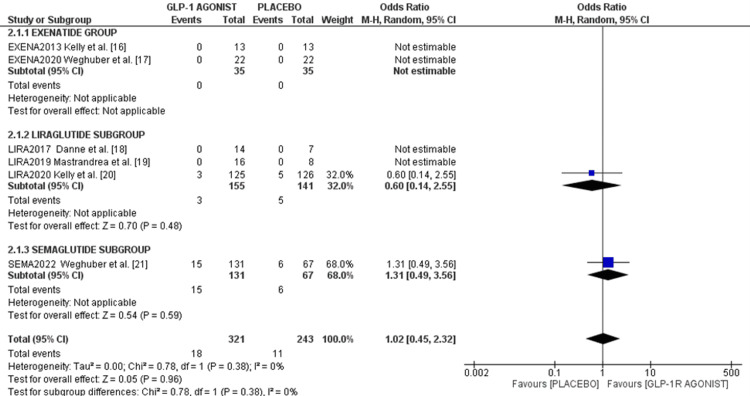
Forest plot of treatment discontinuation rate due to adverse events References: [16-21]

Treatment discontinuation rate due to adverse events: Exenatide and two liraglutide studies did not report treatment discontinuation rates due to adverse events. However, Kelly et al. reported that 13 (10%) participants discontinued due to adverse events with an OR of 30.3 (p = 0.02) [20]. Weghuber et al. reported six (4.5%) versus three (4.4%) participants discontinuing in the semaglutide and placebo arms, respectively, with an OR of 1.02 (95%CI 0.25, 4.23), p = 0.97 [21], as shown in the forest plot in Figure 6. 

**Figure 7:**
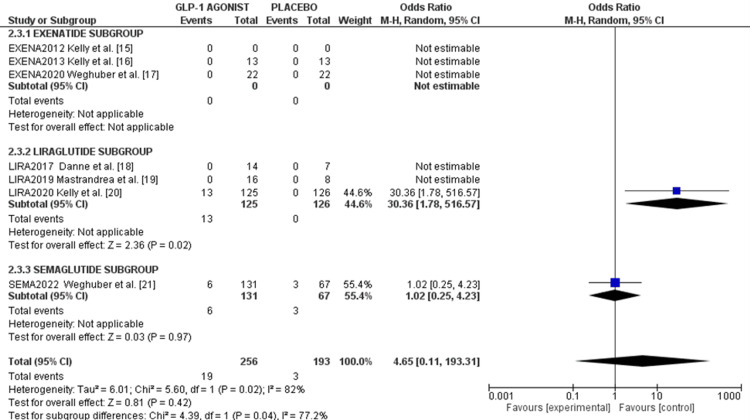
Forest plot of serious adverse events References: [15-21]

SAEs and mortality: Only two studies reported SAEs. Kelly et al.'s study reported three events of SAE (2.4%) in the liraglutide group and five (3.9%) in the control group with an OR of 0.60 (95%CI 0.14, 2.55), p = 0.48. In Weghuber's study, 15 events (11.4%)were reported in the semaglutide arm and six (8.9%) in the placebo arm, with an OR of 1.31 (95%CI 0.49, 3.56), p = 0.59. The pooled analysis showed similar SAE rates between GLP-1 A and placebo groups (1.02 (95%CI 0.45, 2.32), I² = 0%, p = 0.96), as shown in the forest plot in Figure 7.

Risk of Bias in Studies

**Figure 8:**
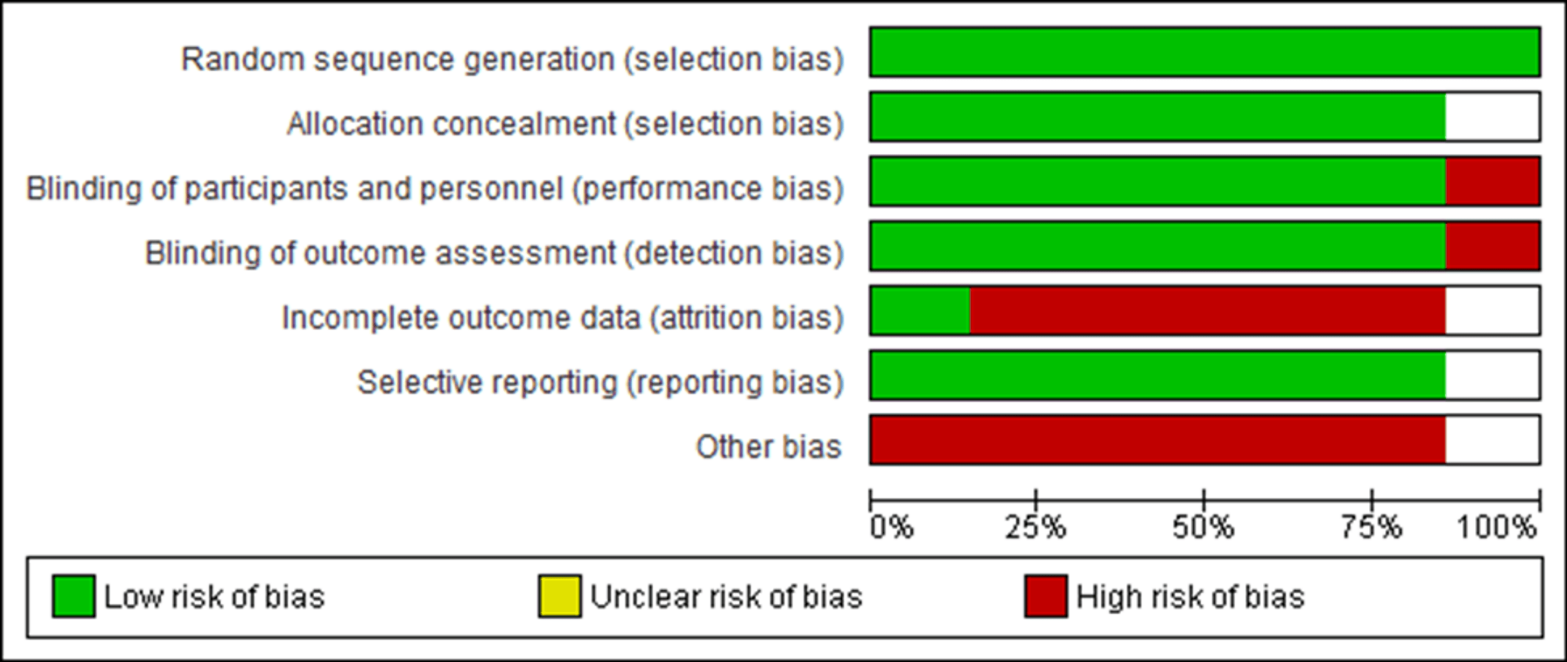
Risk of bias graph

**Figure 9:**
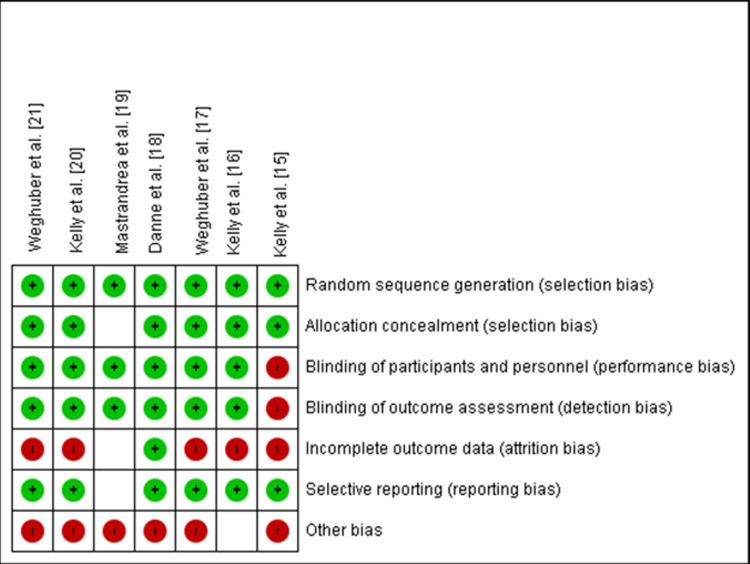
Risk of bias summary

**Figure 10:**
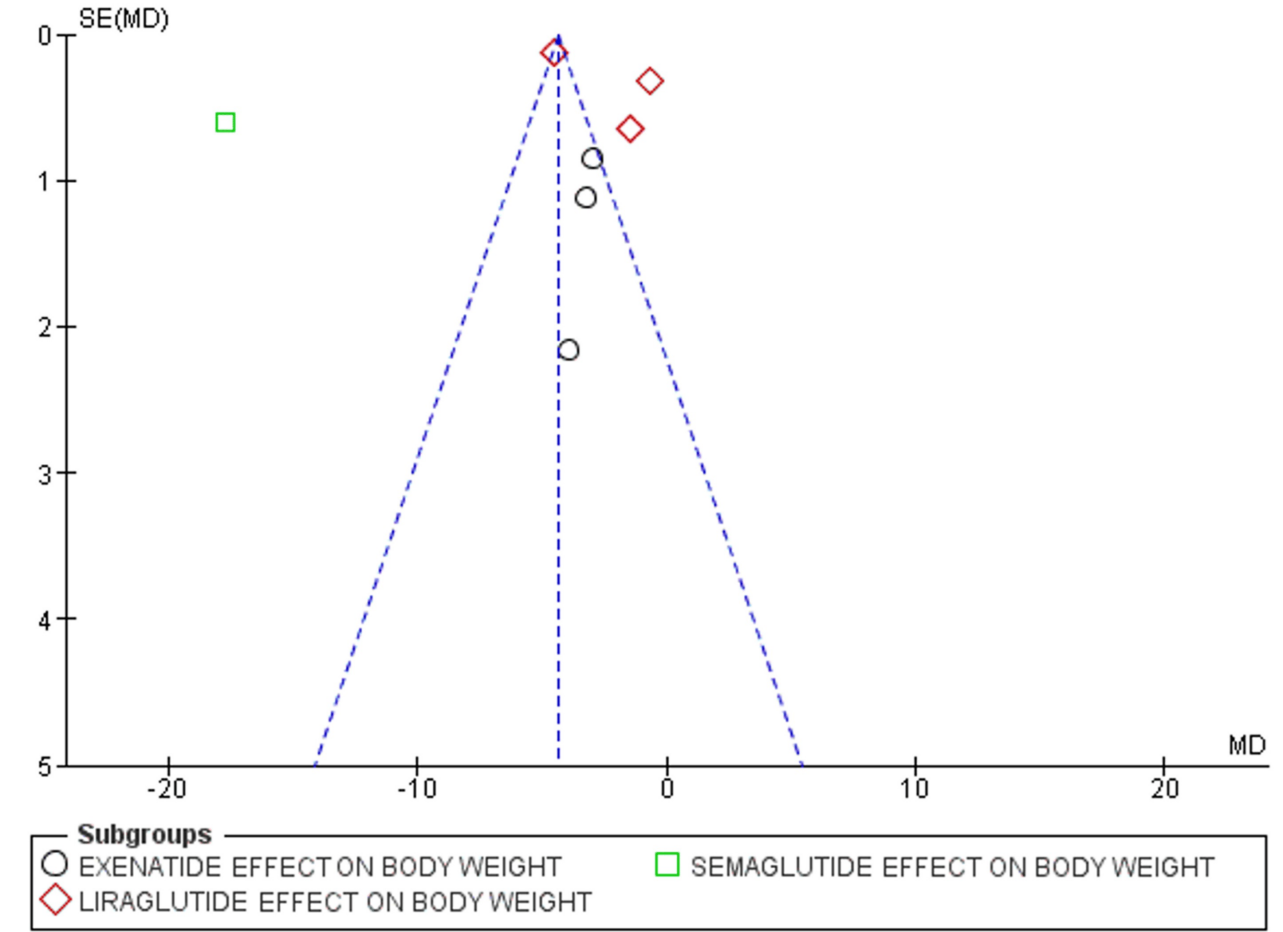
Funnel plot comparing the effect of GLP-1A on body weight SE: standard error; MD: mean difference

While we analyzed studies for risk of bias, all studies were rated as either some concern or high risk as shown in the risk of bias graph in Figure 8, and the risk of bias summary shown in Figure 9. Out of the seven studies, six were fully or partially sponsored by pharmaceuticals. While evaluating publication bias, the funnel plot demonstrated an asymmetrical distribution, indicating publishing bias possibly existed (Figure 10).

Discussion

In this meta-analysis, we evaluated the anti-obesity and safety of GLP-1 analogs in non-diabetic obese or overweight adolescent populations. The meta-analysis included seven RCTs involving a total of 576 children and adolescents, with 333 of them receiving a GLP-1RA. The findings revealed that GLP-1RAs led to significant weight loss in the adolescent population and similar reductions were also seen in BMI and BMI z-scores parameters as compared to the control group. Nevertheless, there was also a strong correlation seen between GLP-1 agonists and gastrointestinal adverse events.

Obesity is an important risk factor for the development of diabetes. According to earlier United States surveys, the risk of diabetes rises by 4.5% and about 9%, respectively, with every kilogram increase in measured weight and self-reported weight [22]. Therefore, managing obesity is critical for preventing or slowing the progression of diabetes. In the study by Hampl et al., a weight loss of 17.7 kg in the semaglutide group was found to be clinically significant; this can lead to substantial improvements in long-term cardiovascular risks related to childhood obesity [23].

GLP-1 receptor analogs play a critical role in anti-obesity treatment. In diabetic patients, they increase insulin secretion and decrease glucagon secretion. They decrease body weight by reducing calorie intake through reduced gastrointestinal tract motility and an anorectic action via activation of GLP receptors in the brain [24]. Our meta-analysis confirmed the significant weight loss action of GLP-1RA in adolescent non-diabetic populations. Similar results were found in a meta-analysis by Chaddah et al., which was performed on the adolescent population [11]. It included nine studies with exenatide and liraglutide as interventions and involved a total of 286 children and showed that GLP-1 analogs reduced overall body weight by -1.86 kg and more significant weight reduction was seen in children with obesity (-2.74 kg) than in children with diabetes (-0.97 kg). Here also, the most common adverse events of GLP-1 RA were gastrointestinal symptoms.

Another meta-analysis by Ryan et al. appraised nine trials involving 574 participants with or without type 2 diabetes mellitus [12]. Studies of exenatide and liraglutide showed modest weight-reducing effects with minor gastrointestinal side effects. They also found no significant difference in the efficacy of liraglutide and exenatide. Unlike these studies, our meta-analysis included semaglutide and focused on overweight or obese children without diabetes.

Similar results are seen while comparing with meta-analyses done on the adult population. A recent meta-analysis by Gua et al. involving 24 studies with 5867 adults without diabetes showed that semaglutide led to a total weight loss of -8.12 kg, liraglutide -5.45 kg, and exenatide -3.23 kg, similar to our results [25]. Another adult meta-analysis by Vogushi et al. reviewed 60 RCTs with 24,969 patients and found the semaglutide intervention reduced the highest proportion of >5% weight loss, followed by liraglutide and exenatide [26].

Semaglutide provides better weight reduction outcomes than other GLP-1AR because of its special effects on controlling appetite, reducing food cravings, and lowering the desire for fatty meals. Semaglutide's metabolism mainly occurs through the enzyme neprilysin, resulting in higher plasma levels than liraglutide, contributing to its more pronounced anti-obesity effect. Also, semaglutide has a higher affinity for the GLP-1 receptor, which enhances its efficacy on GLP-1A receptors [27,28]. Additionally, weekly administration of semaglutide improves patient adherence compared to the daily doses of liraglutide and exenatide [29].

GLP-1R agonists were linked to mild to severe gastrointestinal side effects such as pancreatitis, delayed gastric emptying, nausea, vomiting, constipation, diarrhea, and abdominal discomfort [30]. GLP-1RAs have been associated in recent studies with a higher incidence of intestinal obstruction, gastroparesis, and pancreatitis [31]. All three GLP-1RAs in our meta-analysis showed similar gastrointestinal adverse effect incidences, consistent with previous pediatric meta-analyses, although adult studies reported varying tolerability levels between different GLP-1RAs.

Regarding treatment discontinuation due to adverse events, only two studies reported it. Weghuber et al.'s study found similar discontinuation rates between groups [21], while Kelly et al.'s study reported significantly higher odds of discontinuation due to adverse events in the liraglutide arm [20]. Overall, more extensive clinical trials are needed for clearer insights.

No significant increase in SAEs or all-cause mortality was found across the studies. While analyzing the risk of bias, we found all included studies had some concern or high risk. Allocation bias was seen in the study by Kelly et al. [15], attrition bias was also significant in five studies, and nearly all included studies are pharmaceutical-sponsored trials.

This study's strengths include being the most updated review of GLP-1RAs' effects on the pediatric population, including the more recent GLP-1A semaglutide, and being the first meta-analysis to focus on overweight adolescents without diabetes. This could provide strategies for managing obesity in non-diabetic pediatric populations.

Nevertheless, there are a few limitations, such as the very limited number of studies, high heterogenicity observed between studies, and small sample sizes in the included studies. To develop safe and effective treatments for obese individuals, more high-quality clinical trials are required to assess the weight loss effects of GLP-1RAs, especially semaglutide studies, in pediatric populations.

## Conclusions

This review highlights the effectiveness of GLP-1A, especially semaglutide, in reducing weight as well as BMI in the adolescent obese non-diabetic population while maintaining safety. GLP-1 agonists have great potential to improve the health and quality of life of people with obesity and can prevent obesity-associated complications. However, additional research and long-term studies are necessary to verify the long-term efficacy, safety of semaglutide in this population. Currently, GLP-1 analogs and many other anti-obesity drugs are mostly approved for adolescents aged 12 years and over, limiting pharmaceutical alternatives for youngsters under 12. Considering the increasing rates of childhood obesity under 12 years of age, expanding the use of GLP-1 agonists to younger children could be seen as a potential intervention strategy to halt this serious risk factor for several metabolic syndromes in adulthood.
